# Comparison between an SGLT2 inhibitor and insulin in tumor-to-tissue contrasts in ^18^F-FDG PET imaging of diabetic mice

**DOI:** 10.1038/s41598-023-45094-3

**Published:** 2023-10-26

**Authors:** Liang Meidai, Du Yujing, Liu Zhaoyu, Li Shanshi, Zhao Guangyu, Fan Yan, Yang Xiuying, Zhang Jianhua

**Affiliations:** 1 Department of Nuclear Medicine, Peking University First Hospital, Beijing, 100034 People’s Republic of China; 2 Beijing Key Laboratory of Drug Target and Screening Research, Institute of Materia Medica of Peking, Union Medical College, Beijing, 100050 People’s Republic of China; 3 Department of Nuclear Medicine, Beijing Chaoyang Hospital, Capital Medical University, Beijing, 100020 People’s Republic of China; 4 Department of Radiation Oncology, Peking University First Hospital, Beijing, 100034 People’s Republic of China

**Keywords:** Cancer imaging, Translational research, Endocrine system and metabolic diseases

## Abstract

^18^F-fluorodeoxyglucose positron emission tomography (^18^F-FDG PET) has been widely utilized for tumor diagnosis. Hyperglycemia affects the ^18^F-FDG uptake and reduces tumor-to-tissue contrasts, however, ideal hypoglycemic drugs are lacking. This study compared the role of insulin with the novel widely used hypoglycemic drug, sodium-glucose cotransporter 2 (SGLT2) inhibitor, on ^18^F-FDG PET imaging in diabetic conditions. The streptozotocin (STZ)-induced diabetic C57BL/6N mice were inoculated with B16 (mouse melanoma) cells to establish the xenograft tumor model. After the mice had been administrated with dapagliflozin (30 mg/kg, IG) or insulin (0.75 U/kg, IP) for one hour, 9.25 MBq/10 g ^18^F-FDG was injected. Biodistributions were detected by gamma counting and microPET imaging. The results showed dapagliflozin did not significantly affect the ^18^F-FDG uptake in tumors but reduced uptake in reference tissues, resulting in a significant increase in the tumor-to-skeletal muscle ratio. Conversely, insulin increased ^18^F-FDG uptake in tumors without significant reduction in uptake in reference tissues; Although there was an observable improvement in tumor imaging, it did not reach significantly statistical differences. This study suggests that insulin and SGLT2 inhibitor yield comparable effects on the quality of ^18^F-FDG PET imaging in diabetic patients. Nevertheless, SGLT2 inhibitors would be more suitable when skeletal muscle is used as reference tissue.

## Introduction

^18^F-Fluorodeoxyglucose positron emission tomography (^18^F-FDG PET), in combination with high-resolution anatomical structure detection such as computed tomography (CT) or magnetic resonance imaging (MRI), has been widely used in clinical diagnotics^1–3^. It entails the comparison of ^18^F-FDG uptake in tumors with that in reference tissues to ascertain the presence of tumor cells^2^. However, elevated blood glucose levels can increase ^18^F-FDG uptake in tissues^4,5^. In diabetic patients, hyperglycemia adversely influences the PET detection of multiple tumors by reducing the glucose uptake ratio between tumors and contrast tissues^6^, and consequently diminishes diagnostic accuracy^7^.

Currently, managing fasting blood glucose levels for diabetic patients who undergo ^18^F-FDG PET/CT examination remains a challenge^1,5,8^. Collaborative nuclear medicine groups such as the European Nuclear Medicine Association (EANM) and the American Academy of Radiology (ACR) have proposed protocol guidelines for the management of diabetic patients undergoing ^18^F-FDG PET/CT examination^1^. The current accepted guidance for diabetic patients is if their blood glucose level exceeds 11 mmol/L (approximately 200 mg/dL), the patient must either defer the examination, use rapid-acting insulin, or wait until their blood glucose meets the requirements^1,5^. Since insulin can significantly increase tissue glucose uptake, the interval between insulin and ^18^F-FDG injection should exceed four hours^1^. However, insulin notably influences glucose transporter 4 (GLUT-4), leading to excessive glucose/FDG uptake by skeletal muscle^1^. In addition to the risk of hypoglycemia, insulin can also result in false-negative tumor detection^1,8,9^. Additionally, 9.5% of diabetic patients still had blood glucose levels above 10 mmol/L^8^. Although limited research has been conducted, other oral hypoglycemic drugs, such as sulfonylureas, glinides, dipeptidyl peptidase IV inhibitors, glucagon-like peptide-1 receptor agonists, and thiazolidinediones, which can either directly or indirectly promote insulin secretion or enhance insulin’s effects, potentially leading to nonspecific glucose/FDG absorption by tissues^10^. Metformin, one of the first-line hypoglycemic drugs for type 2 diabetic patients^11^, can increase glucose/FDG uptake in tissues, especially in the intestine, necessitating discontinuation 48 h before the examination^12^. Fasting alone can also enhance the sensitivity of ^18^F-FDG PET examination, but prolonged fasting time inconveniences patients^7,13,14^.

There are two major types of glucose carrier proteins within the body, namely facilitative glucose transporters (GLUTs) and active sodium-glucose cotransporters (SGLTs). Currently, sodium-glucose cotransporter 2 (SGLT2) inhibitors, such as dapagliflozin, are widely used to lower blood glucose levels in diabetic patients, particularly for elderly patients^11,15^, who may have difficulty tolerating extended fasting preparation. This suggests that SGLT2 inhibitors could serve as a potential preparation intervention for ^18^F-FDG PET/CT examination. Unlike the aforementioned hypoglycemic drugs, the hypoglycemic effect of SGLT2 inhibitors is closely linked to the glomerular filtration rate, leading to the elimination of glucose from the body through the urinary system^16^, a pattern that aligns with the excretion of ^18^F-FDG^11^. Studies have demonstrated that SGLT2 inhibitors effectively reduce the blood glucose levels in diabetic patients without significantly impacting the glucose uptake of tissues, and they have no significant effect on tissue insulin sensitivity^11,15^. Malignant tumor cells predominantly utilize overexpressed glucose transporters, especially GLUT1, GLUT3, and SGLT2, for glucose uptake^17–19^. However, the uptake of ^18^F-FDG is nearly unaffected by SGLT2 inhibitors because ^18^F-FDG is primarily a substrate of GLUTs and exhibits lower affinity for SGLTs^11,15,20^.

It has been demonstrated that B16 cells (mouse melanoma cells) can be used to accurately estimate tumor volume in ^18^F-FDG PET imaging^21^. Melanoma is known to express GLUT1 and GLUT3 transporters^22^. Informatic analysis (http://gepia.cancer-pku.cn/detail.php) of SGLT2 gene expression in melanoma revealed that, in line with findings in most malignancies, there are no significant alterations in the expression.

Given the prevailing challenges in clinical ^18^F-FDG-PET imaging for patients with diabetes, this study aimed to compare the effects of SGLT2 inhibitors and insulin, and to provide clues for diabetic patients to choose appropriate hypoglycemic drugs in ^18^F-FDG PET imaging diagnosis.

## Materials and methods

### Animals

In this study, male C57BL/6J mice (20–22 g) were used. The animals were purchased from Beijing Vital River Laboratory Animal Technology Co., Ltd. (Beijing, China). Mice were housed in environmentally controlled conditions with a 12-h light/dark cycle at temperature of 22 ± 3 °C and humidity of 55 ± 5%. Mice were given free access to food and water for seven days before the experiment. This study and all procedures using animals were in accordance with ARRIVE guidelines^23^ and approved by the Animal Care and Use Committee of the Institute of Materia Medica, Chinese Academy of Medical Sciences. All the methods were performed in accordance with the relevant Ethical guidelines and regulations (Ethical committee approval No.00009471).

### Cell culture and viability detection

B16 cell line (mouse melanoma cells) was purchased from FuHeng Biology Co., Ltd. (Shanghai, China). The cells were cultured in high glucose (4500 mg/L) Dulbecco’s modified Eagle’s medium (DMEM) (Thermo) supplemented with 10% FBS at 37 °C in 5% CO_2_ atmosphere.

### STZ-induced diabetic mouse modeling

After fasting for 16 h, diabetes was induced by intraperitoneal injections of streptozotocin (STZ, InnoChem Science & Technology Co., Ltd., Beijing, China) at a dose of 120 mg/kg. Control mice received an equal volume of the vehicle only (citrate buffer, pH 4.5). Seven days after the administration of STZ, 4-h fasting blood glucose levels were measured with an ACCU-CHEK® Active glucometer (Roche, Hoffmann, Germany). The mice whose fasting blood-glucose levels exceeded 11 mmol/L were chosen as the diabetic mouse models.

### Inoculation for tumor xenografts in mice

The xenograft tumor model in mice was established following the procedures reported previously^24^. In brief, after the tumor cells were digested with trypsin and harvested, the cells were suspended in phosphate buffer solution (PBS) (pH 7.2) and kept on ice. Normal or diabetic modeling mice, which were 12-week-old male C57BL/6 mice, were routinely disinfected with 75% ethanol on the right axillary skin, and 0.1 mL of tumor cell suspensions (containing 1.5 × 10^6^ cells) were injected under the axilla, while the control mice were injected with the equal volume of PBS. The body weights of mice and the sizes of tumor grafts were recorded regularly. The tumors were measured using calipers, and the volumes were calculated as [0.52 × (length × width^2^)]. When the tumor grew up to 1000 mm^3^ in size, the animals were subjected to microPET scan and ^18^F-FDG biodistribution.

### Animal groups and drug treatments

After the modeling of tumors combined with diabetes in mice, 4-h fasting blood glucose levels were tested. The tumor combined with diabetes mice were divided into three equal groups (n = 10) according to blood-glucose levels and body weights, including the diabetic tumor control group, dapagliflozin intragastric administration group (30 mg/kg, IG), and insulin intraperitoneal administration group (0.75 U/kg, IP). Euglycemic mice inoculated with tumor xenografts were used as the tumor control mice. Dapagliflozin was purchased from Shanghai Yuanye Bio-Technology Co., Ltd. (Cas 461432–26-8, Shanghai, China), and insulin for injection was purchased from Wanbang Biopharmaceuticals Co., Ltd. (Xuzhou, Jiangsu, China).

### ^18^F-FDG biodistribution detection in mice

Mice were fasted for 2 h, and mice in the administration groups were given drugs by the corresponding routes. One hour later (fasting for a total of three hours), 2-[^18^F]-FDG (radiochemical purity > 95%, atom Hi-Tech Co., Ltd., Beijing, China,) was injected into the tail vein at a dose of 9.25 MBq/10 g body weight (0.25 mCi/10 g). After ^18^F-FDG was distributed in conscious mice for one hour, the mice were anesthetized with isoflurane, and the blood, tumor, and tissues were collected. Gamma counting was performed following tissue weighing. After attenuation correction, the percentage of radiation uptake per gram of tissue (%ID/g) was calculated for each tissue and tumor.

### ^18^F-FDG microPET scan in mice

Mice were imaged using a Mosaic HP Small Animal PET imager (Philips Medical Systems, Inc., Cleveland, Ohio, USA) for ^18^F-FDG PET imaging. The administration of drugs and 2-[^18^F]-FDG in mice was the same as for the biodistribution. ^18^F-FDG was distributed in the mice for 1 h while awake. To prevent movement contamination and confounding the result with ^18^F-FDG FDG uptake in muscles, the anesthetized animalsused here for biodistribution and imaging detection^25^. A mixture of oxygen (95%) and isoflurane (5% for induction and ≤ 2% for maintenance) was used to anesthetize the mice. Then, the mice were gently fixed with the prone position on an imaging bed and subjected to six-minute static PET scanning. The images were scanned, corrected and reconstructed using the ordered subset expectation maximization reconstruction algorithm (ESEM2D) of the MOSAIC HP PET system. Data analysis was performed according to the region of interest (ROI) drawn on the tumor and tissue. uWS-MI software (United Imaging Healthcare Co., Ltd, Shanghai, China) was used for image display and volume of interest (VOI) analysis. The displayed values were the pixel values. For tumors, the mean% and max% (the single largest pixel value in the ROI) were reported to avoid bias in the ROI by including any necrotic tumor areas.

### Statistical analysis

The differences between the groups were tested using unpaired 2-tailed Student’s *t* tests or ANOVAs with Tukey’s post hoc analysis, as appropriate. Data were analyzed using GraphPad Prism (GraphPad Software, USA). Data were presented as the means ± SEM unless otherwise stated. The significance level was set at *P* ≤ 0.05.

### Ethical approval

This study and all procedures using animals were approved by the Animal Care and Use Committee of the Institute of Materia Medica, Chinese Academy of Medical Sciences (No. 00009471).

## Results

### Both the dapagliflozin and insulin can effectively reduce blood glucose in T1D mice

**Figure 1:**
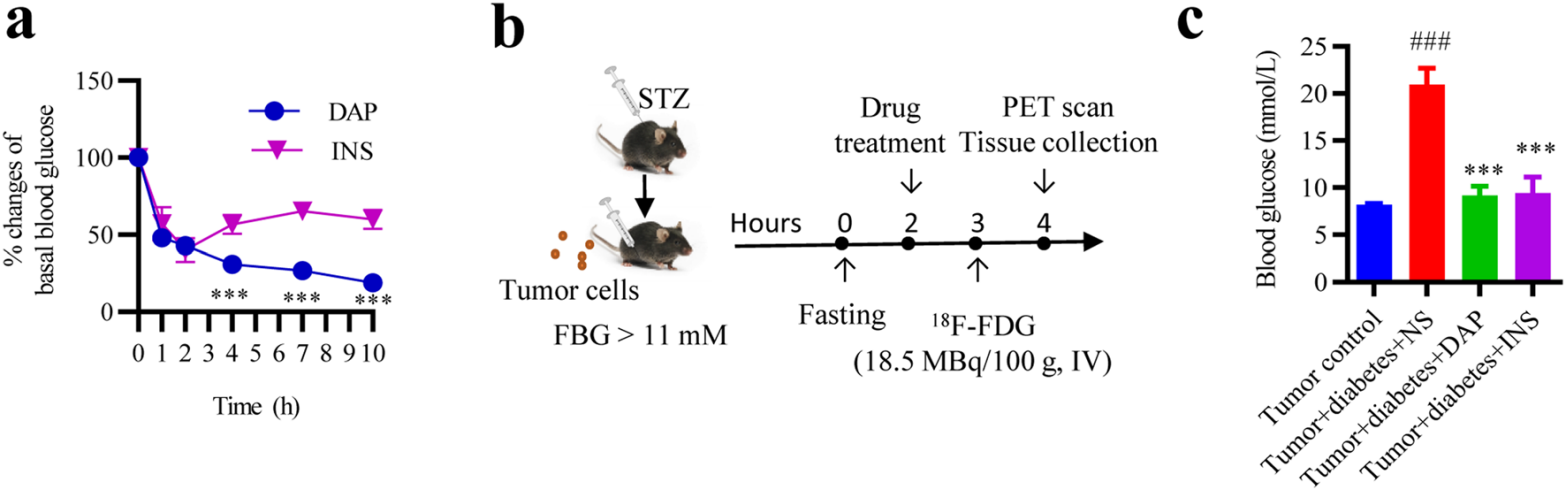
Effects of dapagliflozin and insulin on blood glucose levels in diabetic mice. (**a**) Comparison of the hypoglycemic effects between dapagliflozin and insulin. (**b**) Strategy for studying of dapagliflozin on ^18^F-FDG PET imaging in diabetic mice with tumors. (**c**) Blood glucose levels of each group. Data represent the means ± SEMs, n = 10; biological replicates. ^###^*P* < 0.001 compared with tumor control, ***P < 0.001 compared with tumor diabetic control or indicated group. Statistical analysis was performed by unpaired 2-tailed Student’s t test (**a**) or one-way ANOVA followed by Fisher’s LSD test (**c**).

To clarify the time effect of dapagliflozin and insulin on blood glucose in diabetic animals, dapagliflozin (30 mg/kg, IG) and rapid-acting insulin (0.75 U/kg, IP) were administered to diabetic animals. The results showed that within 2 h after administration, the glucose-lowering effects of both drugs were comparable. However, after three hours, dapagliflozin continued to reduce blood glucose, which was significantly stronger than that of insulin. The optimal hypoglycemic effects were achieved when drugs were administered after 1 h (blood-glucose level below 10 mmol/L) (Fig. 1a). Other studies have found that one hour after the rats^9^ or diabetic patients^26,27^ were given insulin, ^18^F-FDG injection imaging shows a good contrast between tumors and tissues. In present study, dapagliflozin and insulin were administered one hours before ^18^F-FDG injection, and biodistribution were detected another one hour later via gamma counting and microPET imaging. The experimental process is shown in Fig. 1b. Both drugs were effective in lowering blood glucose to comparable levels before ^18^F-FDG injection (Fig. 1c).

### Effects of drugs on ^18^F-FDG uptake in tumor and reference tissues of diabetic mice

**Figure 2:**
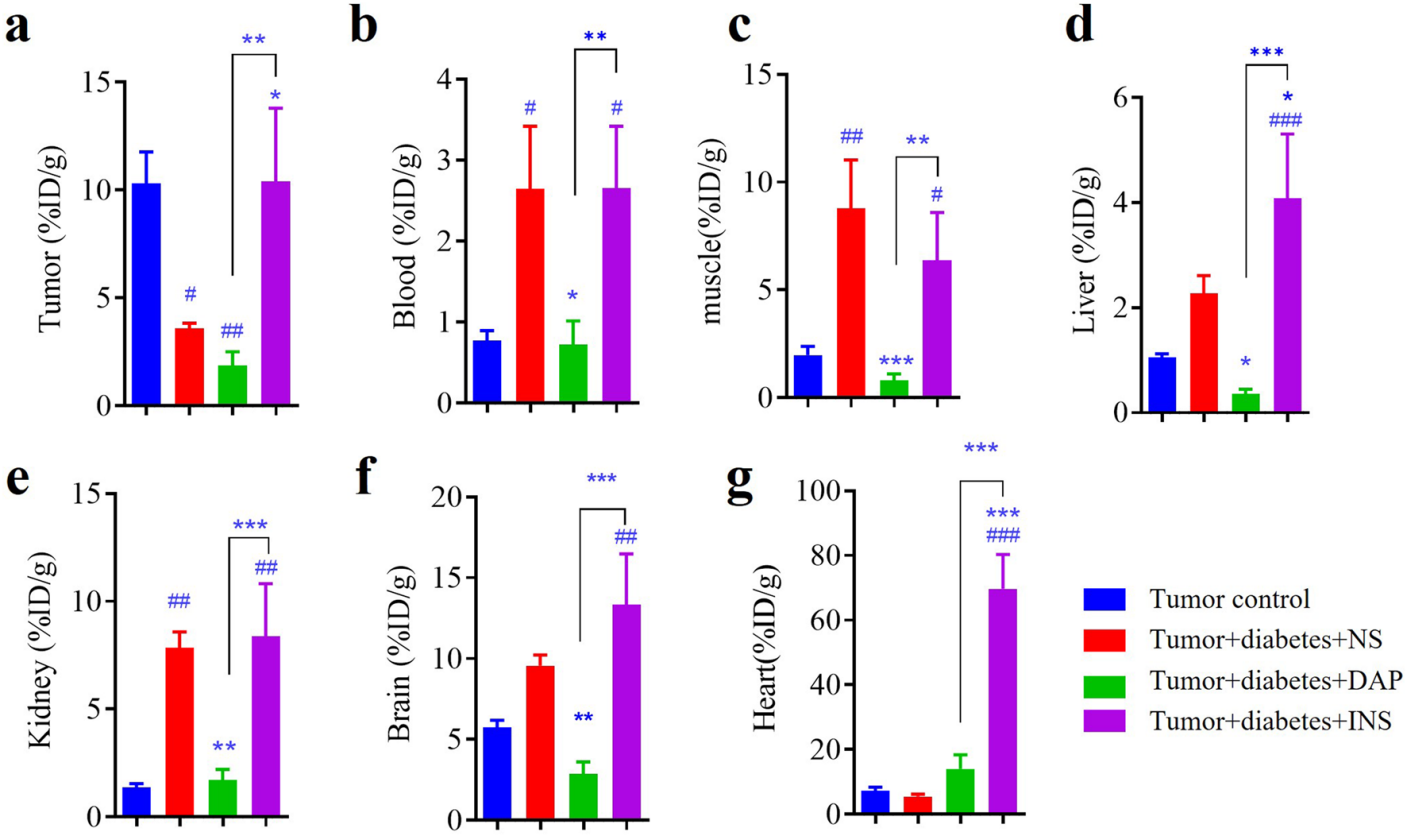
Effects of drugs on ^18^F-FDG uptakes in diabetic mice based on gamma counting. (**a**) Tumor tissue. (**b**) Blood. (**c**) Skeletal muscle. (**d**) Liver. (**e**) Kidney. (**f**) Brain. (**g**) Heart. Data represent the means ± SEMs, n = 10; biological replicates. ^#^*P* < 0.05, ^##^*P* < 0.01, ^###^*P* < 0.001 compared with tumor control, *P < 0.05, **P < 0.01, ***P < 0.001 compared with tumor diabetes control or the indicated group. Statistical analysis was performed by one-way ANOVA followed by Fisher’s LSD test.

This study revealed that diabetes significantly decreased ^18^F-FDG uptake in tumor tissue compared to euglycemic state (Fig. 2a). Diabetic animals exhibited elevated levels of ^18^F-FDG in reference tissues when compared to non-diabetic animals, including blood (Fig. 2b), skeletal muscle (Fig. 2c), liver (Fig. 2d), and kidney (Fig. 2e). Dapagliflozin did not significantly affect ^18^F-FDG uptake in diabetic tumor tissues (Fig. 2a), but prominently reduced ^18^F-FDG levels in blood (Fig. 2b), skeletal muscle (Fig. 2c), liver (Fig. 2d), kidney (Fig. 2e), as well as brain (Fig. 2f). Insulin administration significantly increased ^18^F-FDG uptake in tumor tissue (Fig. 2a), liver (Fig. 2d), and heart (Fig. 2g) in diabetic animals but did not have a significant impact on ^18^F-FDG uptake in blood (Fig. 2b), skeletal muscle (Fig. 2c), kidneys (Fig. 2e), and brain (Fig. 2f). Surprisingly, insulin increased the uptakes of ^18^F-FDG in tumors in diabetic animals, which was a result markedly different from the effects of dapagliflozin administration (Fig. 2a).

### Biodistribution detection results of the ^18^F-FDG uptake ratios between tumor and reference tissues

**Figure 3:**
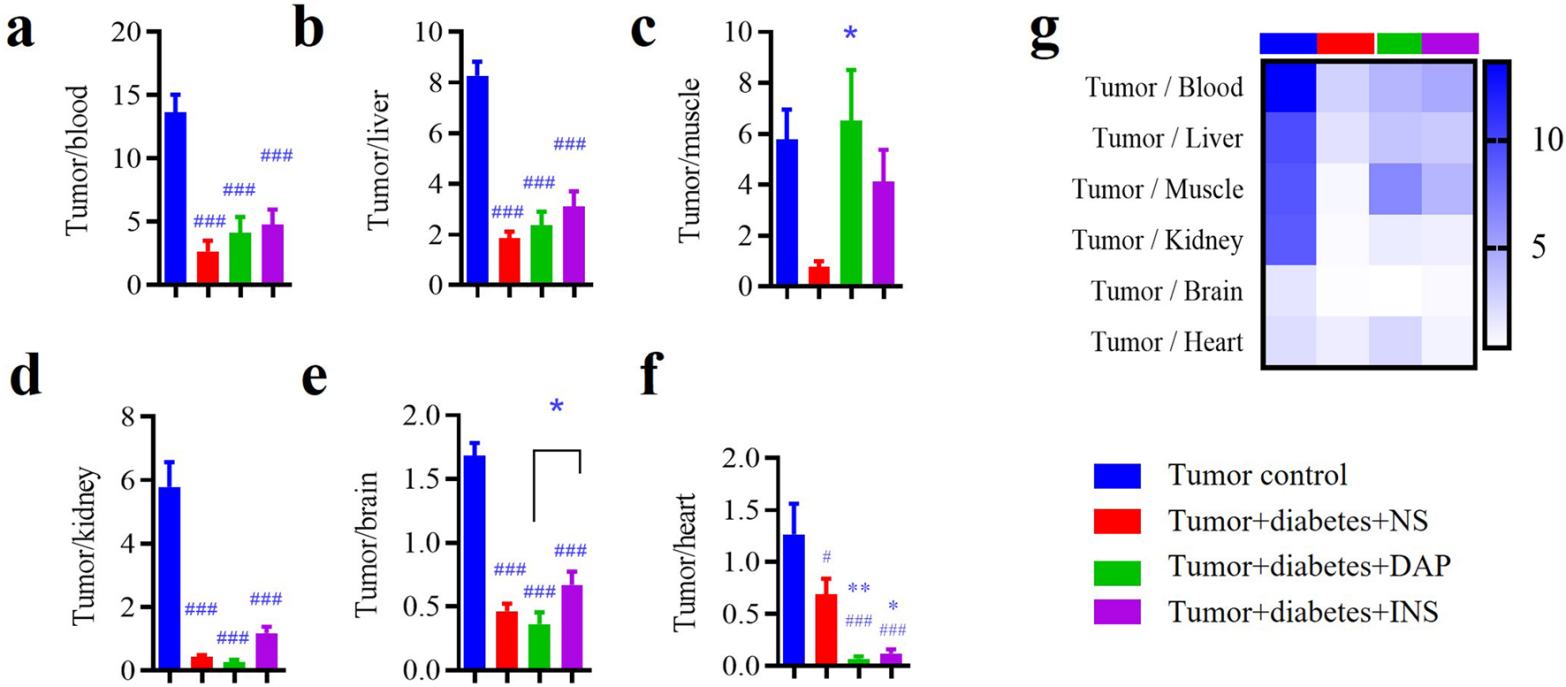
Effect of drugs on tumor-to tissue ratios of ^18^F-FDG uptake by gamma counting. (**a**) Tumor to blood. (**b**) Tumor to liver. (**c**) Tumor to skeletal muscle. (**d**) Tumor to kidney. (**e**) Tumor to brain. (**f**) Tumor to heart. (**g**) Heatmap of the ^18^F-FDG uptake ratios among groups. Data represent the means ± SEMs, n = 10; biological replicates. ^###^*P* < 0.001 compared with tumor control, *P < 0.05, **P < 0.01 compared with tumor diabetes control or the indicated group. Statistics analyzed by one-way ANOVA followed by Fisher’s LSD test.

This study demonstrated the ^18^F-FDG tumor-to-tissue uptake ratios were ranked in descending order as blood, liver, skeletal muscle, kidney, brain, and heart under euglycemic condition (Fig. 3a–g). In diabetic conditions, ^18^F-FDG tumor-to-tissue uptake ratios decreased, meanwhile the order remained similar to that of euglycemic mice (Fig. 3g). Dapagliflozin administration apparently increased the tumor-to-skeletal muscle ratio (Fig. 3c), which was comparable to that of normal diabetic animals. The ratios in dapagliflozin-administered group were ranked from high to low as skeletal muscle, blood, and liver (Fig. 3g), while the order of ratios was blood, skeletal muscle, and liver in insulin treated group (Fig. 3g). Insulin administration did not significantly increase ^18^F-FDG uptake ratios of tumor to reference tissues in diabetic mice (Fig. 3a–f).

### Effects of the two drugs on ^18^F-FDG PET imaging in diabetic mice

**Figure 4:**
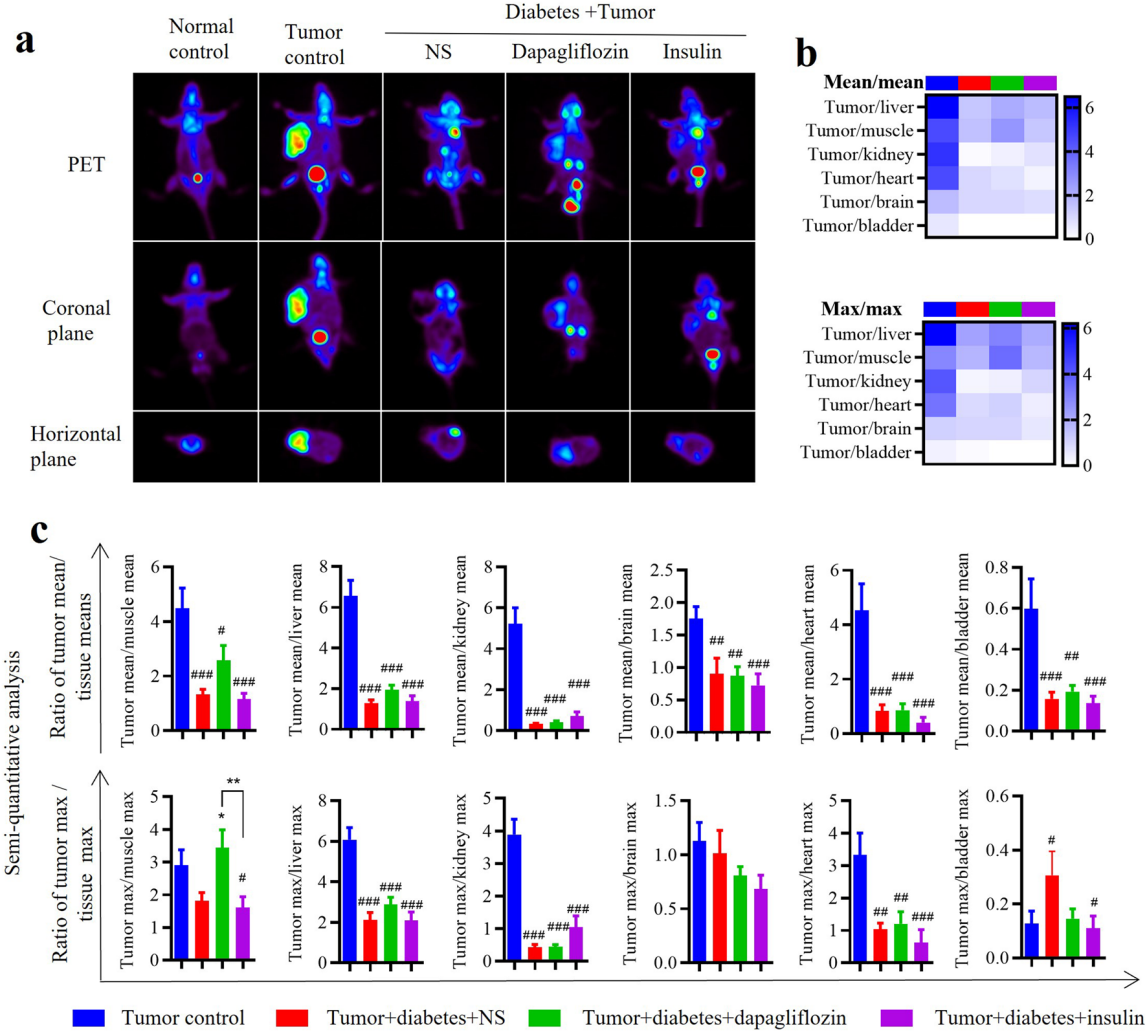
Effect of dapagliflozin and insulin on ^18^F-FDG PET imaging in diabetic mice. (**a**) Representative photos of ^18^F-FDG PET examination. (**b**) Heatmap of the ^18^F-FDG PET semiquantitative analysis results of tumor mean% to reference tissue mean% (upper), and tumor max% to reference tissue max% (lower). (**c**) Ratio of tumor mean% to reference tissue mean% and tumor max% to reference tissue max%. Data represent the means ± SEMs, n = 7–10; biological replicates. ^#^*P* < 0.05, ^##^*P* < 0.01, ^###^*P* < 0.001 compared with tumor control, *P < 0.05 compared with tumor diabetes control or the indicated group. Statistical analysis was performed by one-way ANOVA followed by Fisher’s LSD test.

The results of PET semiquantitative analysis manifested diabetic condition played an important role in increasing ^18^F-FDG imaging pixels in various reference tissues (Fig. 4a). The semiquantitative analysis results of PET showed similar ranking of tumor-to-tissue ratios among different groups as observed in biodistribution detection (Fig. 4b). Dapagliflozin increased the ^18^F-FDG uptake imaging contrast of tumor to skeletal muscle of diabetic animals (Fig. 4c). Considering the possibility of central necrosis in tumor that could affect the results, the ratios of tumor mean% pixel and max% pixel to reference tissues were calculated (Fig. 4b, 4c). Comparatively, the results of max% pixels aligned more closely with the findings of biodistribution assay.

## Discussion

This study is the first to discover the SGLT2 inhibitor can effectively reduce ^18^F-FDG uptake in skeletal muscle and significantly increase the tumor-to-skeletal muscle ratio in diabetic mice compared with insulin.

The present work found that the FDG uptake in tumor tissues of diabetic mice decreased, but in reference tissues increased, which is consistent with the results previously reported^9^. In addition, we found that the SGLT inhibitor had no obvious effect on ^18^F-FDG level in tumor tissue, which is in agreement with a previous report that SGLT1/2 has little influence on the transfer of ^18^F-FDG to tumor cells^11^.

Compared to diabetes controls, we also discovered that insulin promoted ^18^F-FDG uptake in B16 tumor tissue. Although insulin can increase glucose uptake in various tissues^1^, the effect of insulin on ^18^F-FDG uptake in tumor tissue under diabetic conditions remains to be elucidated. An in vitro study showed there was no significant change of ^18^F-FDG uptake in human adenocarcinoma cells with chronic hyperglycemia (300 mg/dL), while acute hyperglycemia markedly reduced ^18^F-FDG uptake^28^. Studies have found in the mammary carcinoma of STZ-induced diabetic rats, after 1 h of insulin administration, insulin didn't affect the FDG uptake of tumor, the uptake ratio of tumor to liver and blood increased, but to skeletal muscle decreased^9^. One study reported that insulin reduced FDG uptake in allogenic hepatoma cells (KDH-8) in euglycemic rats^29^. Clinical studies show that insulin administration one hour before ^18^F-FDG injection neither improves imaging quality^27^ nor affect ^18^F-FDG uptake in lung cancer tissue of diabetic patients^26^. In present study, we found that the imaging of ^18^F-FDG PET showed an improvement trend with insulin treatment, but without statistically significant differences. Insulin stimulates the up-regulation of GLUT4, resulting in the transfer of FDG distribution to peripheral muscle, soft tissue and fat, with a low ratio of tumor targeting to reference tissues uptake^30^. Although insulin has no obvious effect on the expression of transcription factors other than GLUT4, it can induce the transfer of GLUT1^31^, GLUT3^32^ to cell membranes. Melanoma has also been reported to express GLUT4^33^. Thus, we speculate that the increased uptake of insulin-promoted FDG in B16 melanoma may be related to the above factors, which requires further verification.

Generally, GLUTs are the main transporters for 2-FDG entry into tumor cells, yet, 2-FDG is also a substrate of SGLTs^20^. Studies have reported that glucose uptake in some types of tumor cells is dependent on SGLTs^34^. SGLT2 is expressed in the early development of lung cancer, specifically in precancerous lesions and well-differentiated adenocarcinoma^18^. In cases of SGLT2 overexpression and lower GLUT1 expression, false-negative outcomes may occur in certain tumor metastases^35^. Considering that SGLT2 gene expression levels are lower than GLUTs in most solid tumors^36^, and ^18^F-2-FDG is not a specific substrate of SGLT2, we speculate the use of hypoglycemic SGLT2 inhibitors would be more beneficial in ^18^F-FDG PET diagnosis than insulin for most patients with hyperglycemia combined with tumors.

Importantly, our study found that when the SGLT2 inhibitor was used, the blood ^18^F-FDG levels significantly decreased compared with both the diabetic control and insulin groups, which has important implications for clinical application. Although SGLTs have far lower affinity for FDG than glucose^37^, approximately 10% of FDG is reabsorbed by the proximal tubule of the kidney^38,39^. In diabetic state, SGLT2 expression is elevated in the proximal tubule^40^. Therefore, we infer the reasons for the lower ^18^F-FDG levels in reference tissues, including blood and tissues, may be due to the inhibition of SGLT2, thereby promoting its excretion, whereas insulin has no effect in this regard.

Excitingly, this study uncovered a novel effect, unlike the effect of insulin, SGLT2 inhibitors can dramatically reduce ^18^F-FDG uptake in diabetic skeletal muscle. Decreasing the ^18^F-FDG uptake of reference tissues under hyperglycemia is a crucial way to enhance the imaging contrast of tumor tissue for ^18^F-FDG PET detection. Previous studies have reported that SGLT2 inhibitors could effectively reduce the blood-glucose level of diabetic patients without significantly increasing the glucose uptake of tissues^11,15^, yet our study showed that SGLT2 inhibitors decreased the glucose/FDG uptake in reference tissues, especially in skeletal muscle. It is noteworthy that the similar uptake in tumor tissues and reduced uptake in nontarget tissues also suggeste that SGLT2 inhibitors may be have beneficial for patient by reducing the radiation load to organs, and decreasing the harmful radiation toxicity.

The work concurrently has several limitations. One issue is that a study has shown diabetic state increases the expression of renal tubular SGLT2^40^. Therefore, the impact of diabetes on the expression of SGLTs and GLUTs in tumors and reference tissues may affect ^18^F-FDG imaging and need to be clarified when SGLT2 inhibitors administered. Additionally, the influence of SGLT2 inhibitors on the half-life of ^18^F-FDG in the blood is also a consideration. Dapagliflozin reaches its peak within two hours after entering the bloodstream and has a half-life of 12.9 hours^41^. Different SGLT2 inhibitors also have different half-lives and hypoglycemic effects, so researches on the time and dose effect of SGLT2 inhibitors on ^18^F-FDG tissue uptake and elimination will further guide clinical applications. Furthermore, more clinical studies are needed to observe and validate whether the results of this study can be applied to humans.

## Conclusion

This study is the first to investigate the impact of SGLT2 inhibitor on ^18^F-FDG PET imaging and provides a comparative analysis with the effects of insulin. In this study, we have unveiled that SGLT2 inhibitors exhibit the capacity to notably reduce ^18^F-FDG uptake in skeletal muscle compared with that of insulin under hyperglycemic condition, and enhance the tumor-to-skeletal muscle contrast. This study implies that SGLT2 inhibitors may be beneficial for ^18^F-FDG PET diagnostic in diabetic patients with skeletal muscle as the reference tissue. However, further clinical validation is still needed.

## Data Availability

The datasets used or analyzed during the current study are available from the corresponding author on reasonable request.

## Abbreviations

CT: Computed tomography
^18^F-FDG: ^18^F-fluorodeoxyglucose
GLUT: Glucose transporters
IG: Intragastric administration
IP: Intraperitoneal administration
MRI: Magnetic resonance imaging
PET: Positron emission tomography
SGLT2: Sodium-glucose co-transporter 2
STZ: Streptozotocin
T1D: Type 1 diabetes
